# Strategies for integrating animal social learning and culture into conservation translocation practice

**DOI:** 10.1098/rstb.2024.0138

**Published:** 2025-05-01

**Authors:** Alison L. Greggor, Shermin de Silva, Culum Brown, Brett R. Jesmer, Daniel W. A. Noble, Thomas Mueller, Carlos R. Ruiz-Miranda, Christian Rutz, Sarah Elizabeth Scott, James Williams

**Affiliations:** ^1^ Conservation Science Wildlife Health, San Diego Zoo Wildlife Alliance, Escondido, CA, USA; ^2^ Ecology Behavior and Evolution, University of California San Diego, La Jolla, CA, USA; ^3^ Trunks and Leaves, Pittsfield, MA, USA; ^4^ School of Natural Sciences, Macquarie University, Sydney, New South Wales, Australia; ^5^ Department of Fish and Wildlife Conservation, Virginia Tech, Blacksburg, VA, USA; ^6^ Division of Ecology and Evolution, Research School of Biology, ANU College of Science and Medicine, Canberra, Australian Capital Territory, Australia; ^7^ Senckenberg Gesellschaft fur Naturforschung, Frankfurt am Main, Hessen, Germany; ^8^ Department of Biological Sciences, Goethe University Frankfurt, Frankfurt, Hessen, Germany; ^9^ Laboratório de Ciências Ambientais, Universidade Estadual do Norte Fluminense Darcy Ribeiro, Campos Dos Goytacazes, Rio de Janeiro, Brazil; ^10^ Centre for Biological Diversity, School of Biology, University of St Andrews, Scotland UK; ^11^ Joint Nature Conservation Committee, Peterborough, Cambridgeshire, UK

**Keywords:** captive-to-wild translocation, conservation breeding, conservation behaviour, non-human culture, reintroduction, wild-to-wild translocation

## Abstract

Conservation translocations are increasingly used in species’ recovery. Their success often depends upon maintaining or restoring survival-relevant behaviour, which is socially learned in many animals. A lack of species- or population-appropriate learning can lead to the loss of adaptive behaviour, increasing the likelihood of negative human interactions and compromising animals’ ability to migrate, exploit resources, avoid predators, integrate into wild populations, reproduce and survive. When applied well, behavioural tools can address deficiencies in socially learned behaviours and boost survival. However, their use has been uneven between species and translocation programmes, and behaviour commonly contributes to translocation failure. Critically, current international guidance (e.g. the International Union for Conservation of Nature’s translocation guidelines) does not directly discuss social learning or its facilitation. We argue that linking knowledge about social learning to appropriate translocation strategies will enhance guidance and direct future research. We offer a framework for incorporating animal social learning into translocation planning, implementation, monitoring and evaluation across wild and captive settings. Our recommendations consider barriers practitioners face in contending with logistics, time constraints and intervention cost. We emphasize that stronger links between researchers, translocation practitioners and wildlife agencies would increase support for social learning research, and improve the perceived relevance and feasibility of facilitating social learning.

This article is part of the theme issue ‘Animal culture: conservation in a changing world’.

## 1. Introduction

Conservation translocation is an umbrella term that covers the human-mediated movement of animals for conservation purposes. Translocations encompass wild-to-wild transfers of animals and the release of animals bred in captivity into the wild [1], as well as transfers from the wild to captivity and between captive populations for conservation breeding purposes. The success of translocations depends on animals being able to find food and shelter, avoid predators and reproduce [2]—behaviours that in many species are either known or suspected to rely on social learning. Social learning is the acquisition of information from the actions or cues left by others, which can lead to stable behavioural differences between groups of conspecifics—a phenomenon known as animal culture (for definitions and a methodological toolkit for studying these phenomena, see [3]). Very few translocation programmes explicitly consider animal social learning and the cultural variants that can emerge from it, as neither are mentioned in recent reviews of translocation outcomes (e.g. [4–6]), in inventories of translocation strategies [7] or in widely used translocation guidelines [1]. Despite the lack of attention culture has received, there are mechanistic reasons why social learning processes and resulting behaviours should affect translocation outcomes [8–12]. For example, the act of translocation can disrupt existing social groupings and release animals into habitats where they may be ill-equipped to interact with novel selection pressures or conspecifics [10,11,13,14]. Yet, given that social learning can be challenging to pinpoint [3], post-translocation issues stemming from social learning are rarely reported and may be simply viewed as post-release behavioural deficits exhibited by translocated individuals, which are common [4]. For instance, without adequate opportunities for social learning, animals can lose adaptive migratory behaviour (e.g. whooping cranes, *Grus americana* [15]; bighorn sheep, *Ovis canadensis* [16]; lesser spotted eagles, *Clanga pomarina* [17]) and struggle to acquire resources (e.g. adequate food in carnivores [18]), especially in seasonal or changing environments where resource distributions are spatially and temporally variable [19,20].

When translocated animals lose socially learned foraging patterns and knowledge of resource availability, they may become more reliant on easy-to-find, ‘cheap’ resources (e.g. food, shelter), which can lead to negative human–wildlife interactions (e.g. bears, Ursidae [21,22]), and impact translocation outcomes. Continued conflict and translocation failure are also increasingly likely when translocation is undertaken to relocate ‘conflict individuals’ away from human-dominated landscapes [23–25]. The low efficacy of these mitigation translocations may be partially due to socially learned behaviours or their absence since detrimental interactions with humans are often socially learned (e.g. black bears, *Ursus americanus* [26]; bottlenose dolphins, *Tursiops aduncus* [27]).

Issues stemming from social learning in translocated animals can also impact how they interact with resident populations. The capacity of animal populations to absorb translocated individuals may depend not only on their density and local environmental conditions but also on the demographics of the recipient population and the willingness of conspecifics to interact and engage with new arrivals [10]. Integration may depend on age, sex, familiarity [28], relatedness or species’ life history (e.g. age at dispersal, social organization and mating system [10]), but may also be hindered by a mismatch in socially learned behaviour. For instance, differences in the mode, content or specific variants of communication—many of which are socially learned—can influence fitness, interactions and post-release survival [29,30]. The impacts of reduced integration can be far-reaching; when released animals remain isolated from residents or experience excess mortality, they lower the reproductive contributions of release efforts. Lesser reproduction or greater mortality reduces the conservation benefit of translocations and increases the resources and number of releases necessary for achieving translocation goals. Additionally, large releases of culturally mismatched individuals also have the potential to negatively impact recipient populations, for instance by flooding them with novel traits that can interfere with, ‘pollute’ (e.g. [31]), or cause the extinction of locally adaptive behaviours (e.g. [32–34]).

There can also be long time lags between cultural disturbances and their observable consequences, making it hard to detect issues. For instance, cultural groups of sperm whales (*Physeter macrocephalus*) have fitness advantages that emerge during El Niño weather cycles only every 3–5 years [35], and elephant (*Loxodonta*) group knowledge about water resources may only be relevant for fitness once every few decades [36]. Moreover, since social learning can be challenging to document and quantify empirically [3], these issues are likely to be under-reported in the peer-reviewed literature [37] and under-appreciated in wider translocation discourse. Perhaps for these reasons, issues pertaining to animal social learning and culture are rarely identified or studied systematically with respect to translocation outcomes (e.g. [5,6]), which limits practitioners’ access to potentially helpful ways of predicting outcomes and improving success. Yet, there are a number of tactics practitioners can take to avert the disruption of social information during translocation.

**Figure 1.**
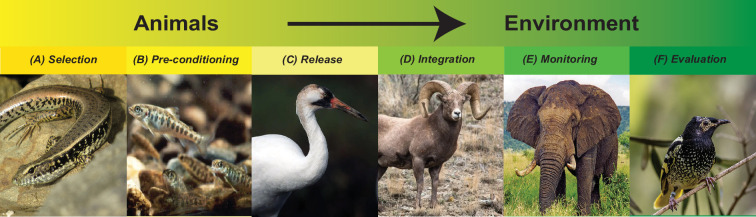
Examples of the stages of a translocation programme where social learning or culture can be used. Translocations involve a series of key steps that include selection of suitable animals and environments, pre-conditioning animals, animal release, monitoring and evaluation [7]. Culture and social information use can be considered at any stage in the pathway to successful translocation. (*A*) Selection of animals is an important decision. Age and social status are known to influence the propensity for social learning. For example, young lizards (*Eulam prus quoyii*) are known to learn where and on what to forage from adult conspecifics [38], which could improve foraging success of translocated animals in new locations if released together. (*B*) Prior to release, it is often necessary to train captive-reared animals to respond appropriately to stimuli they are likely to encounter in the wild. For example, hatchery-reared Atlantic salmon (*Salmo salar*) can be taught to forage for live prey in appropriate locations [39], and species ranging from birds to small mammals can benefit from social learning about predators [13]. (*C*) The social composition of released animals can facilitate integration with existing animals and maximize information transfer among group members. For example, whooping cranes migrate more effectively when migrating with older birds [15]. (*D*) Released animals may also benefit from having opportunities to acquire socially learnt information about the environment. For example, bighorn sheep translocated to environments where the species had been extirpated lacked knowledge necessary to migrate, but sheep translocated to existing populations socially learnt how to migrate [40]. Such considerations could be important for deciding where translocation would be most appropriate. (*E*) Monitoring of translocated animals in their environment should take place to understand what socially relevant behaviours may be important for translocation success. For example, elephant social structure has been shown to impact the occurrence of aberrant, potentially problematic behaviours in young males, which are rectified through the introduction of older bulls [41]. Such intervention requires monitoring of social behaviour. (*F*) Social manipulations before and after translocations should be evaluated for their effectiveness, and the cost–benefit of considering social information should be explored. For example, evaluating the success of captive release programmes on the survival of translocated regent honeyeaters revealed that song tutoring using wild individuals was important for increased survival post-release [30]. Image credits: (*A*) John Tann, (*B*) Peter E. Steenstra/USFWS, (*C*) Ryan Hagerty/USFWS, (*D*) public domain, (*E*) Caitlin, TheLizardQueen and (*F*) Jss367.

Social learning can be either explicitly staged, controlled or considered when engaging in the majority of documented strategies for conducting conservation translocations (table 1; electronic supplementary material, table S1). These actions can conserve diverse types of cultural behaviours and can be tailored to target diverse taxa (figure 1). For example, bird song culture can be maintained by exposing juvenile birds to appropriate playbacks of their species’ song during rearing (e.g. regent honeyeaters, *Anthochaera phrygia* [30]). Migratory behaviour can be maintained by ensuring released animals have opportunities to interact with and follow knowledgeable residents (e.g. in fish, [32]). Socially learned information about foraging skills, or dangers, such as predators, can be spread by giving animals opportunities to observe others (e.g. socially facilitated anti-predator training benefits prairie dogs *Cynomys ludovicianus* [42], and surrogate rearing enhances foraging and survival in southern sea otters, *Enhydra lutris nereis* [43]). The range of potential applications is vast, but the field lacks widespread guidance on where and how to employ strategies that consider or use socially learned information or aspects of cultural variation [11], and when to prioritize directing resources to conserving culturally important behaviour. Research that ties learning outcomes to translocation success is critical. Moreover, since animal social learning has mostly been an academic discipline rather than an applied science, finding and accessing the necessary information to employ effective strategies is also challenging [44].

**Table 1.** Areas where social learning or culture could be used as a tool to influence translocation outcomes (list of wider translocation strategies adapted from [7]). An expanded table, with examples where available, is provided as electronic supplementary material, table S1.

strategy	considerations	meaning	purpose
selection	behavioural, demographic, genetic, physiological and experiential selection criteria.	selection of individuals or groups from pool of candidates based on any of the listed attributes.	accounts for individual-level attributes that might be relevant for cultural learning.
preconditioning	behavioural, physiological, social, experiential preconditioning.	alteration of any of these attributes within individuals or groups prior to release through exposure or training.	provides opportunities to prepare target individuals with key competencies in each domain prior to release.
release design	population size, genetic composition, demographic composition, social composition.	controlling any of these specified attributes of the translocated cohort.	accounts for how the number and variation among individuals could influence key competencies.
environmental selection	suitability, similarity.	selection of an environment based on the level of suitability to the translocated wildlife, as well as similarity to those of the source populations.	considers potential for negative interactions (e.g. conflict) and local adaptations.
environmental preconditioning	pre-release resource augmentation and threat control.	augmentation of resources and control of threats within the recipient environment prior to release.	facilitates appropriate resource use based on socially acquired knowledge and reduces potential risks.
environmental release design	spatio-temporal configuration, release timing, manner of release (delayed or immediate).	control of the number and configuration of release sites, events, timing and extent of holding period.	accommodates the potential for social learning both in terms of spatial and temporal factors
post-release population management	interventions, managed dispersal.	actions undertaken to mitigate issues based on post-release observations and establish/maintain meta-population dynamics.	applies social learning markers or cues to identify and address problems, as well as influence movements.
post-release environmental management	post-release resource augmentation and threat control.	augmentation of resources and control of threats within the recipient environment following release.	facilitates ongoing learning about resources and decreases risk until behavioural competencies are acquired.

Here, we present a framework for integrating aspects of social learning and culture into translocation practice to facilitate their explicit recognition, study and effective use. We consider the structure of translocation decision-making and present benefits alongside caveats and barriers to application. We also offer a perspective on how current translocation guidance can be augmented with social learning strategies.

## 2. Steps for application

**Figure 2.**
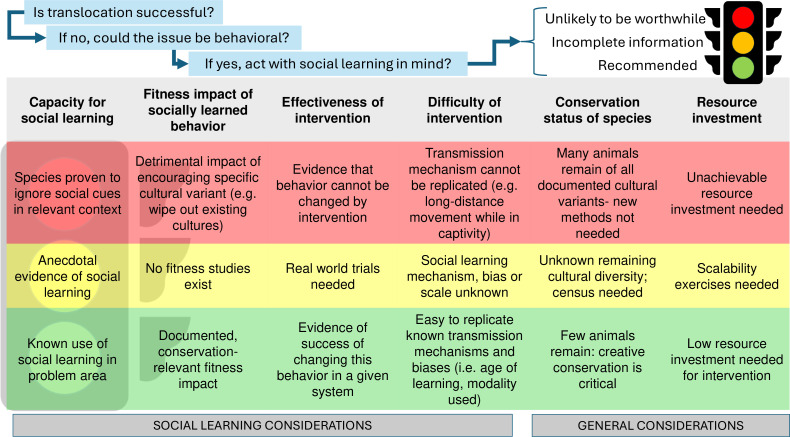
Decision support for pursuing interventions that target social learning in translocation programmes. If a programme identifies that behaviour may be negatively impacting success, there are several avenues (columns of text) to consider before embarking on an intervention that targets social learning. For each avenue, there may only be incomplete information available, highlighting uncertainty about the outcome. Programmes need to decide—based on a careful cost–benefit analysis—whether to proceed in light of this uncertainty or to pursue research to resolve it (yellow light). Green lights suggest that, at least along that avenue, the intervention should benefit from applying social learning and cultural processes. However, if any avenue hits a red light, this is an indication not to proceed. The ultimate value of any avenue must be weighed with the context of the wider programme in mind. For instance, although a documented fitness benefit to a cultural behaviour may exist, if that may only be realized once in a lifetime (e.g. to help during multi-decade drought), it may be considered less important than behaviours that accrue benefits more predictably or over shorter timescales (such as every year). The temporal scales over which cultural factors operate are in need of further study, which translocations may provide the opportunity to test in some cases. During translocation planning, it may be worthwhile to conduct this decision support exercise while considering the potential problem behaviours that might arise. Different values may be placed on each avenue depending on the specific behaviour and species.

Identifying where social learning and culture impact behaviour is pertinent to many topics within this special issue (i.e. [3,9,45]). However, before delving into these details, a more pressing question for many managing conservation projects is whether to alter their intervention at all. Regardless of the potential relevance of social learning and culture to a translocation effort, there can be significant time, logistical and monetary constraints that make it infeasible or inadvisable to adjust strategies. Herein, we outline a series of decision-making avenues that highlight when it might be advisable to pursue interventions that target social learning (figure 2). For example, many projects may see the potential value of pre-release training for a socially learned behaviour (e.g. [46,47]), but if doing so is logistically challenging and there is a very low likelihood that it will increase translocation success, then this may not be a good investment of limited time and resources. Assessing the value of applying social learning to a project can reveal knowledge gaps about whether a given behaviour is learned socially or how scalable an intervention may be from a theoretical laboratory setting to the complex, often messier, context of a translocation. These unknowns can magnify the risk of an intervention or make it challenging to quantify its benefits.

Practitioners need to contend with the logistics and feasibility of the many non-behavioural aspects of a translocation effort, such as land agreements, infrastructure, animal transport, etc. Funding limitations are also a common challenge for translocation projects [4]. Therefore, it is useful to identify where considering social learning is cost-effective compared with existing protocols [44]. In salmonids, for instance, the time, effort and cost involved with considering social learning by training anti-predator and foraging behaviour pays off since it sufficiently enhances post-release survival [46]. Sometimes small changes to translocation protocols can enhance the potential for the social transfer of behaviour. For instance, timing releases to coincide with natural dispersal or other social learning opportunities. Alternatively, for some species, simple and inexpensive changes to housing (e.g. socializing captive-born individuals with wild-born ones) may be all that is required to foster social learning. Conversely, behaviours will need to be acquired post-release in some species. Animals with large home ranges may need to be trained at the release site, especially for long-distance movement behaviours (e.g. human-led migration in whooping cranes [15], or northern bald ibis, *Geronticus eremita* [48]), which can be a challenging endeavour. Other behaviours may be infeasible to replicate in human care (e.g. seasonal movements or responses to weather) and may have to be nudged or seeded via a series of smaller scale manipulations. Additionally, there may only be narrow windows of time or very precise locations where individuals have opportunities to learn certain behaviours (e.g. nest selection or mate choice), which may occur outside of the release timeframe. Post-release, translocated individuals are naive to risks in their new environment and may be prone to picking up behaviours from conspecifics that may harm them. For example, juvenile elephants released into populations where they observe frequent crop foraging by adult elephants will also acquire this behaviour, and thereby become vulnerable to human retaliation [49]. Therefore, practitioners should consider how much they can influence learning outcomes in such scenarios. Research that quantifies the cost or logistical hurdles surrounding existing versus social-learning-focused interventions alongside their relative effectiveness would help guide practice [11].

Practitioners may also want to minimize effort going towards conserving socially learned behaviour that is not relevant to fitness or translocation outcomes. Some socially learned traits may be ‘neutral’, having no measurable impact on survival or reproductive rates, or could even have a negative fitness impact when adopted in a novel environment (e.g. in the absence of new social information, birds favouring man-made nesting platforms may have difficulties expanding to areas without platforms). Meanwhile, there is a risk that practitioners could inadvertently seed non-natural or maladaptive behaviours, potentially corrupting existing cultures if released populations interact with wild ones (e.g. disrupting tool-using behaviour of New Caledonian crows, *Corvus monduloides* [50]; or mistiming the start of migrations with large releases of hatchery-reared fish that attract wild ones to follow). While such risks are theoretically possible and are likely to exist for translocation programmes, additional research is necessary to clarify how likely it is that inadvertent seeding impacts fitness in the wild. More likely, disrupting existing behavioural norms or the emergence of new socially learned behaviours can contribute to unpredictable translocation outcomes. In animals that readily learn from others, managers should be prepared for increased behavioural flexibility post-release (in primates [51]; see also [52,53]) and potential surprises. For instance, when juvenile California condors (*Gymnogyps californianus*) were reintroduced without opportunities to learn habitat preferences and foraging skills from adults, they unexpectedly adopted preferences for dangerous human structures (i.e. power poles) previously unexploited by the species, which risked the failure of the decades-long breeding and translocation effort [54]. These issues were resolved after the birds were brought back into human care, housed with adult tutors and conditioned to avoid power pole structures. Continued social transmission of naturalistic foraging has been facilitated by releasing juveniles near supplementally provisioned food sites that attract adult birds and allow for social interaction post-release [54–56].

Depending on the species, the type of translocation may influence the importance of considering social learning and culture in planning efforts. Translocations can be classified in several ways (*sensu* [1]). For example, ‘reinforcement’ or ‘supplementation’ when conspecifics already occur at a release site, ‘reintroduction’ when the species has been extirpated, ‘assisted colonization’ when habitat may be suitable even if not historically occupied, and ‘ecological replacement’, when the area has lost a comparable species that previously fulfilled an ecological niche. Translocations also happen routinely for conservation breeding purposes in the form of transfers between captive populations and as part of collections from the wild to human care in order to create or bolster breeding populations. Additionally, translocations occur in place of culling animals due to their negative interactions with people or destruction of their habitat due to human development or natural disaster (i.e. ‘mitigation translocations’, such as [57]). In each of the above scenarios, the availability and quality of social information available pre- and post-transfer will differ. Locally appropriate, socially learned behaviours may have been lost in reintroductions or may never have existed historically (e.g. in assisted colonization scenarios), such that animals may need to learn and establish their culture afresh. In theory, better outcomes would be predicted in supplementation/reinforcement contexts, since their release area already contains a repository of (cultural) knowledge (e.g. translocated marine reef fish can learn locations of new feeding grounds when local fish are present; [58]). However, when conspecifics are already present, differences between introduced individuals and residents may lead newcomers to remain distinct and experience reduced reproductive success, higher mortality [59], or even inadvertently propagate detrimental behavioural tactics, such as those that underlie human–wildlife conflict [24,60]. Additionally, the experiences and enrichment offered to animals in conservation breeding populations will determine how behaviourally well-equipped they are to face the challenges of the wild [61], including how they respond to humans and other animals in human-dominated landscapes. Therefore, the planning process for translocations would benefit from considering whether appropriate social information exists for the species in question (figure 1).

More research is needed for understanding how social information flows between animals in the context of translocations, such as between released and resident animals or between connections forged in pre-release contexts (see [11] for a list of relevant research questions). The current state of the field is highly skewed towards certain species and domains. For example, strategies and methods for controlling social learning in salmonids or song learning in passerines are much better understood than the drivers of migration in thousands of other fish species [32], the vocal behaviour of many cetaceans [62], or seasonal migration in ungulates [63]. As this special issue highlights, many species may have the capacity for social learning, but formally demonstrating this is challenging, and obtaining compelling evidence for culture is more challenging still [3].

## 3. Integrating culture into existing guidelines

In cases where interventions targeting social learning are relevant, appropriate and logistically feasible, there are many opportunities within the pipeline of traditional translocation practice to intervene. Yet, for the field at large, there is still a surprising lag in uptake. Despite the development of behavioural techniques aimed at improving translocation outcomes [13,47], their rollout has been inconsistent across species and translocation programmes. For instance, pre-release food training was used in only 21% of animal translocation efforts analysed in a recent review, and soft release methods, such as training, were more common in birds than mammals or reptiles [64]. Consequently, behaviour is still reported as a major reason for translocation failures [4,14]. Current IUCN guidelines [1], the internationally recognized standard for translocation practice, do not currently directly address the role social learning and culture can play in translocations. Since the guidelines define the standards of practice used by multiple regional and national governmental agencies (e.g. in the US they are used by the California Department of Fish and Wildlife and the U.S. Fish and Wildlife Service, to name just a few), updates to the guidelines have great potential to influence translocation policy and success within states and nations. Additionally, there is scope for updates to influence transnational policy, as a recent report prepared for the Scientific Council of the Convention on Migratory Species (CMS) specifically recommended greater alignment with IUCN on issues relating to animal social learning and culture [65]. Updates to the guidelines (discussed below) must have taxonomically broad scope because, although species-specific guidelines exist, they are unlikely to be developed for all of the thousands of species that may require translocation for recovery.

The current set of translocation guidelines [1] only cursorily consider animal behaviour, let alone the learning processes that can underpin it. Where animal behaviour is discussed, they only briefly recommend considering the behavioural ecology of the translocated species, the behaviour of individuals when selecting a release cohort, and monitoring behaviour post-release. The guidelines mention the potential for newly released animals to learn survival skills from wild conspecifics and to face issues with social integration (Annex 7), with no details on specifics or related interventions. The only interventions relevant to social learning suggested in the guidelines are the possibility of cross-fostering within a species to encourage the transmission of relevant behaviours (Annex 7), and for using pre-release behavioural training where needed. However, there is no mention of social learning or tactics that target social learning in any of the sections on candidate selection, release criteria, environmental considerations or monitoring. We propose a number of specific updates to the guidelines (electronic supplementary material) and outline some of the higher level improvements here.

Updates to the IUCN guidelines (e.g. §5.1 and Annex 5) concerning biological feasibility and design would benefit from expanding beyond the genetic considerations of founders (such work would benefit from considering common sampling biases in behavioural research, as covered by the STRANGE framework, [66]) and explicitly incorporating a dedicated treatment of criteria for release-candidate selection and pre-release behavioural training. There is additional scope for extending sections that already acknowledge the potential for socially learned behaviour. For instance, §5.1.4 only makes general recommendations that founders show ‘behaviour’ that is ‘appropriate’ in comparison with wild populations. The need for founders to be adequately socialized and trained through exposure to conspecifics remains unstated and warrants its own discussion, apart from the current subsections that are limited to ecological, physical and genetic considerations. Section 5.2 concerns social acceptance of translocations, and Annex 3 discusses when translocation is an acceptable option, both need to consider the capacity for acquisition of relevant behaviours and minimization of problem behaviours, both with respect to the target species, as well as the human populations they may encounter. Annex 8 discusses post-release behavioural monitoring that could be expanded to include multiple indicators to assess whether animals have acquired locally appropriate foraging, reproductive and social behaviours.

IUCN guidelines also only consider wild-to-wild and captive-to-wild translocations, not transfers of animals between captive facilities or guidelines for collection of animals from the wild. Since candidates for translocation may be born in captivity or pass through human care at various stages of their preparation for release into the wild, the guidelines would benefit from adding an annex explicitly considering such scenarios. Knowledge acquisition at critical developmental stages may need to be mirrored in pre-release conditions, especially in captivity. For instance, giving young animals in conservation breeding programmes access to wild-born tutors or the social cues wild animals generate, such as wild-sourced bird song, can improve survival post-release [30] and *ex situ* breeding success. Some, but not all, of the new guidelines for specific taxa recognize these issues more explicitly. For instance, the guide for the rehabilitation and re-introduction of Asian elephants [49] mentions social learning and offers a structured means of evaluating and learning from prior outcomes that could be reflected in the general guidelines (i.e. adaptive management). Additionally, the amphibian-specific guidelines [67] touch on behavioural tools for promoting learning, such as anti-predator training, and also acknowledge the need for greater research connecting learning outcomes to post-release survival, which is universally an issue. Other guides, such as those for gibbons [68] have no specific mention of culture or socially transmitted behaviour, but discuss releasing young animals with older ones or inserting them into wild groups.

When developing species-specific guidelines, there are a series of questions that can help shape the most relevant social learning approaches for practitioners. These considerations help determine what cues and social contexts need to be available to foster animal learning and are intended to complement other relevant decision support tools (e.g. figure 1, in this article; fig. 2 from [11]). Species differ in how they acquire and process social cues [69]. If the intent is to plant artificial social cues, practitioners must ask what are the main perceptual modalities the species uses to learn socially (e.g. visual, chemosensory, auditory)? Social learning can also be biased by demographic, developmental and individual factors [70–72], which can have implications for enacting social learning interventions in conservation [12]. Are there sensitive periods when learning is likely to occur or temporal periods where social integration may be more likely (e.g. dry season when food availability is low and competition is high)? Are cues only available seasonally? If so, are there ways for the translocated individuals to acquire knowledge of where seasonal resources are and which areas are to be avoided? If live tutors are needed, it is necessary to know whether suitable peers are present and accessible at the release site or in human care. Given that some species require area-specific knowledge (e.g. migration routes or resource locations), what behaviours must be learned and tailored with the prospective release site in mind? Species-specific guides can also include different suggestions depending on the type of translocation. For instance, if wild-to-wild translocation is the goal, are the individuals being moved ‘problem animals’? If so, are they likely to seed problematic behaviour at the new site? And how experienced are human neighbours in coping with such problematic behaviour?

## 4. Ongoing challenges

Despite the wide variety of strategies that could influence or track socially learned behaviours (table 1, electronic supplementary material), many practitioners are likely to face barriers to doing so (figure 2). Practitioners commonly lack sufficient experimental data or evidence to guide species-specific decision-making [73]. Overcoming the lack of basic knowledge about the social learning tendencies of many species requires greater communication between academics and conservation practitioners to help fill information gaps and incorporate new research into translocation planning. Animal culture may also be seen as irrelevant for translocations by wildlife authorities and agencies, given that it has largely been an academic topic, and because existing evidence is often inaccessible to practitioners. However, there has been recent progress in some international agreements, such as efforts by the Convention on the Conservation of Migratory Species to elevate issues surrounding animal culture more broadly in conservation (discussed in [8,9]). Biases may also exist about the relevance of social learning for species that are not group living, despite evidence that even relatively ‘non-social’ species can benefit from greater social contact [74] and from social learning [75]. The amount of social contact may merely predict the speed rather than prevalence of social learning [76]. For example, despite being both aggressive and territorial, juvenile Atlantic salmon still learn by observing conspecifics [77], as do many reptiles, which are thought to be rather asocial (e.g. [60,78,79]).

Conservation actors often face rushed timelines and limited resources, requiring quick decisions to be made sometimes with limited information. However, in cases where the consideration of social learning and culture are likely to be pivotal to the success or failure of a programme, assessing the necessity and feasibility of adjusting strategies is warranted (figure 2). Just as decision-making in the face of uncertainty is part of the skill set for many practitioners, and is in itself a studied discipline [80], there may be lessons learned in using these frameworks for the application of social learning in translocation scenarios, with adaptive management in mind. Additionally, there may be instances where documenting animal culture may not be necessary for defining translocation success, so long as animals can acquire the positive fitness-related behaviours they need, and do not acquire new deleterious ones. Cases where social learning can be assumed, but not proven, may be sufficient for action, as similar levels of precaution have been advocated in working on social learning in broader conservation contexts to prevent inaction (i.e. applying the precautionary principle, [9]).

Ultimately, increasing the overall uptake of non-human culture in conservation practice involves several interrelated approaches [44], including understanding the limits of cultural interventions and designing research with realistic applications in mind. As this special issue highlights, there is growing evidence that animal social learning and culture influence behaviour and fitness in diverse species. The practical application of these findings has yet to be fully realized, but we outline where and how social learning can be integrated into translocation guidelines and planning. Greater appreciation of the role socially learned behaviours can play alongside research to support their realistic application should increase opportunities to improve translocation outcomes.

## Data Availability

Supplementary material is available online [81].
